# The Protective Role of Immunomodulators on Tissue-Type Plasminogen Activator-Induced Hemorrhagic Transformation in Experimental Stroke: A Systematic Review and Meta-Analysis

**DOI:** 10.3389/fphar.2020.615166

**Published:** 2020-12-15

**Authors:** Yang Ye, Yu-Tian Zhu, Hong-Xuan Tong, Jing-Yan Han

**Affiliations:** ^1^ Department of Integration of Chinese and Western Medicine, School of Basic Medical Sciences, Peking University, Beijing, China; ^2^ Tasly Microcirculation Research Center, Peking University Health Science Center, Beijing, China; ^3^ Department of Traditional Chinese Medicine, Peking University Third Hospital, Beijing, China; ^4^ Department of Urology, Peking University Third Hospital, Beijing, China; ^5^ Institute of Basic Theory for Chinese Medicine, China Academy of Chinese Medical Sciences, Beijing, China

**Keywords:** tissue-type plasminogen activator, stroke, meta-analysis, immunomodulator, hemorrhagic transformation, animal model

## Abstract

**Background:** Recanalization with tissue plasminogen activator (tPA) is the only approved agent available for acute ischemic stroke. But delayed treatment of tPA may lead to lethal intracerebral hemorrhagic transformation (HT). Numerous studies have reported that immunomodulators have good efficacy on tPA-induced HT in ischemic stroke models. The benefits of immunomodulators on tPA-associated HT are not clearly defined. Here, we sought to conduct a systematic review and meta-analysis of preclinical studies to further evaluate the efficacy of immunomodulators.

**Methods:** The PubMed, Web of Science, and Scopus electronic databases were searched for studies. Studies that reported the efficacy of immunomodulators on tPA-induced HT in animal models of stroke were included. Animals were divided into two groups: immunomodulators plus tPA (intervention group) or tPA alone (control group). The primary outcome was intracerebral hemorrhage, and the secondary outcomes included infarct volume and neurobehavioral score. Study quality was assessed by the checklist of CAMARADES. We used standardized mean difference (SMD) to assess the impact of interventions. Regression analysis and subgroup analysis were performed to identify potential sources of heterogeneity and evaluate the impact of the study characteristics. The evidence of publication bias was evaluated using trim and fill method and Egger’s test.

**Results:** We identified 22 studies that met our inclusion criteria involving 516 animals and 42 different comparisons. The median quality checklist score was seven of a possible 10 (interquartile range, 6–8). Immunomodulators improved cerebral hemorrhage (1.31 SMD, 1.09–1.52); infarct volume (1.35 SMD, 0.95–1.76), and neurobehavioral outcome (0.9 SMD, 0.67–1.13) in experimental stroke. Regression analysis and subgroup analysis indicated that control of temperature and time of assessment were important factors that influencing the efficacy of immunomodulators.

**Conclusion:** Our findings suggested that immunomodulators had a favorable effect on tPA-associated intracerebral hemorrhage, cerebral infarction, and neurobehavioral impairments in animal models of ischemic stroke.

## Introduction

Currently, thrombolysis with tissue plasminogen activator (tPA) remains the only approved drug treatment for acute ischemic stroke (Pena et al., 2017). However, tPA must be administered intravenously within 4.5 h of ischemic stroke onset due to the increased risk of hemorrhagic transformation (HT) (Mao et al., 2017). HT is believed as one of the leading causes of death and disability after stroke (Xu et al., 2017). Therefore, it is urgent to decrease the risk of HT caused by delayed tPA treatment.

Evidence indicated that inflammatory injury plays an important role in tPA-induced HT (Li et al., 2018). Blood brain-barrier (BBB) damage is the most critical factor in the pathogenesis of HT. Although the mechanism underlying BBB damage is not fully understood, excessive neuroinflammation is thought to be involved in the process (Sorby-Adams et al., 2017). Therefore, immunomodulation seems like a promising direction of drug development for tPA-associated HT. Substances that regulate the function of the immune system are called immunomodulators. Although it is not yet entirely clear how immunomodulators work, it is hypothesized that immunomodulators act on certain points of the immune activation pathways to regulate inflammatory process. They may act as immunosuppressants by inhibiting the immune response or as immunostimulants by stimulating the immune response. A lot of immunomodulators, such as high-mobility group box 1 (HMGB1) inhibitor (Chen et al., 2020) and regulatory T cells therapy (Mao et al., 2017), have been used to relieve HT induced by tPA thrombolysis in animal studies. Although many immunomodulators have shown protective effects on tPA-associated HT, the efficacy of immunomodulators has not yet been systematically reviewed.

In this study, we presented a systematic review and meta-analysis of data from animal studies testing the efficacy of immunomodulators on tPA-induced HT. We aimed to comprehensively review the protective effects of immunomodulators on intracerebral hemorrhage, infarct size, and neurobehavioral outcome in animal models of tPA-induced HT. The factors that influencing the efficacy of immunomodulators in preclinical studies were also identified. Our results may lead to refinements of animal experiments in this field and hence reduce animal numbers required.

## Methods

### Search Strategy

Electronic search was performed in PubMed, Web of Science, and Scopus electronic databases (by July 2020). Studies that reported the efficacy of immunomodulators on tPA-induced HT in animal models of stroke were included. Animals were divided into two groups: immunomodulators plus tPA (intervention group) or tPA alone (control group). The predetermined primary endpoint was intracerebral hemorrhage, and the secondary endpoints included infarct volume and neurobehavioral score. The following search term was constructed to identify animal studies that examined the efficacy of immunomodulators on tPA-induced HT (tPA OR rtPA OR t-PA OR rt-PA OR tissue plasminogen activator OR tissue-plasminogen activator OR alteplase) AND (hemorrhagic transformation OR hemorrhage OR hemorrhage OR bleeding) AND (stroke OR ischemia OR cerebral OR brain). The search strategy is specified in Supplemental Material.

### Inclusion Criteria

Studies were included if they fulfilled the following criteria: 1) the study reported the efficacy of immunomodulators on tPA-induced HT in animal models of stroke; 2) a control group receiving vehicle or no treatment in animal models of tPA-induced HT was described; 3) intracerebral hemorrhage was quantified as an outcome (including hemoglobin content, hemorrhage volume/area/score, studies that only quantified the incidence of HT were excluded); 4) the number of animals per group was described; 5) studies were published in English. Studies were screened by two independent investigators (YY and YTZ) with discrepancies resolved through discussion.

### Data Extraction

We abstracted from studies the publication details (author, year), animal used (sex, species), type of stroke model, intervention used (route, dose, and timing), anesthetic used, tPA administration (dose, timing), and details of the outcome measures. We also extracted the sample size, mean value, and standard deviation for both intervention and control groups. Infarct size was quantified as infarct volume, infarct area, or infarct score. Neurobehavioral outcome was quantified as various neurological scoring system.

If data were only represented graphically, numerical values were extracted using ImageJ software (NIH, Bethesda, MD, United States). When multiple groups were served by a single control group, sample size of the control group was divided by the number of treatment groups (McCann et al., 2014). When outcomes were measured at more than one time point, only data from the latest time point was included. When multiple indicators were used to measure intracerebral hemorrhage, we chose hemoglobin content as our preferred indicator because it is more accurate.

### Quality Assessment

We assessed the quality of the individual publication using the 10-item checklist of CAMARADES (Collaborative Approach to Meta-Analysis and Review of Animal Data in Experimental Stroke) (Sena et al., 2007) comprising the following: 1) publication in a peer-reviewed journal, 2) control of temperature, 3) random allocation to groups, 4) allocation concealment (blinded induction of ischemia), 5) blinded assessment of outcome, 6) use of an anesthetic without intrinsic neuroprotective activity (ketamine), 7) the use of co-morbid animals, 8) performing a sample size calculation, 9) compliance with animal welfare regulations, and 10) statement of potential conflicts of interest. The PRISMA (Preferred Reporting Items for Systematic Reviews and Meta-Analyses) guidelines (Satapathy et al., 2020) were also followed to perform this systematic review and meta-analysis. The study quality was evaluated independently by two researchers (YY and HXT).

### Data Analysis

For each endpoint, we used the standardized mean difference (SMD) effect size to standardize the results to a uniform scale. For intracerebral hemorrhage and neurobehavioral outcome, SMD values were pooled in a weighted mean difference meta-analysis using a fixed-effects model. For infarct size, we combined the comparisons using random-effect meta-analysis. When the pooled SMD effect size (including pooled 95% CI) was greater than 0, it can be defined as an improvement. Heterogeneity across studies was assessed by the Cochran’s Q statistic and quantified by the I2 statistic (Yang et al., 2012). We used meta-regression and subgroup analysis to explore the possible source of heterogeneity. We also used meta-regression to evaluate the impact of the study characteristics. Funnel plots, trim and fill method (Pimpin et al., 2019), and Egger’s test (Wang et al., 2019) were employed to assess the publication bias. We performed sensitivity analysis to confirm the stability of the results. Statistical analyses were performed using Review Manager 5.3 and STATA 13 software.

## Results

### Study Characteristics

**FIGURE 1:**
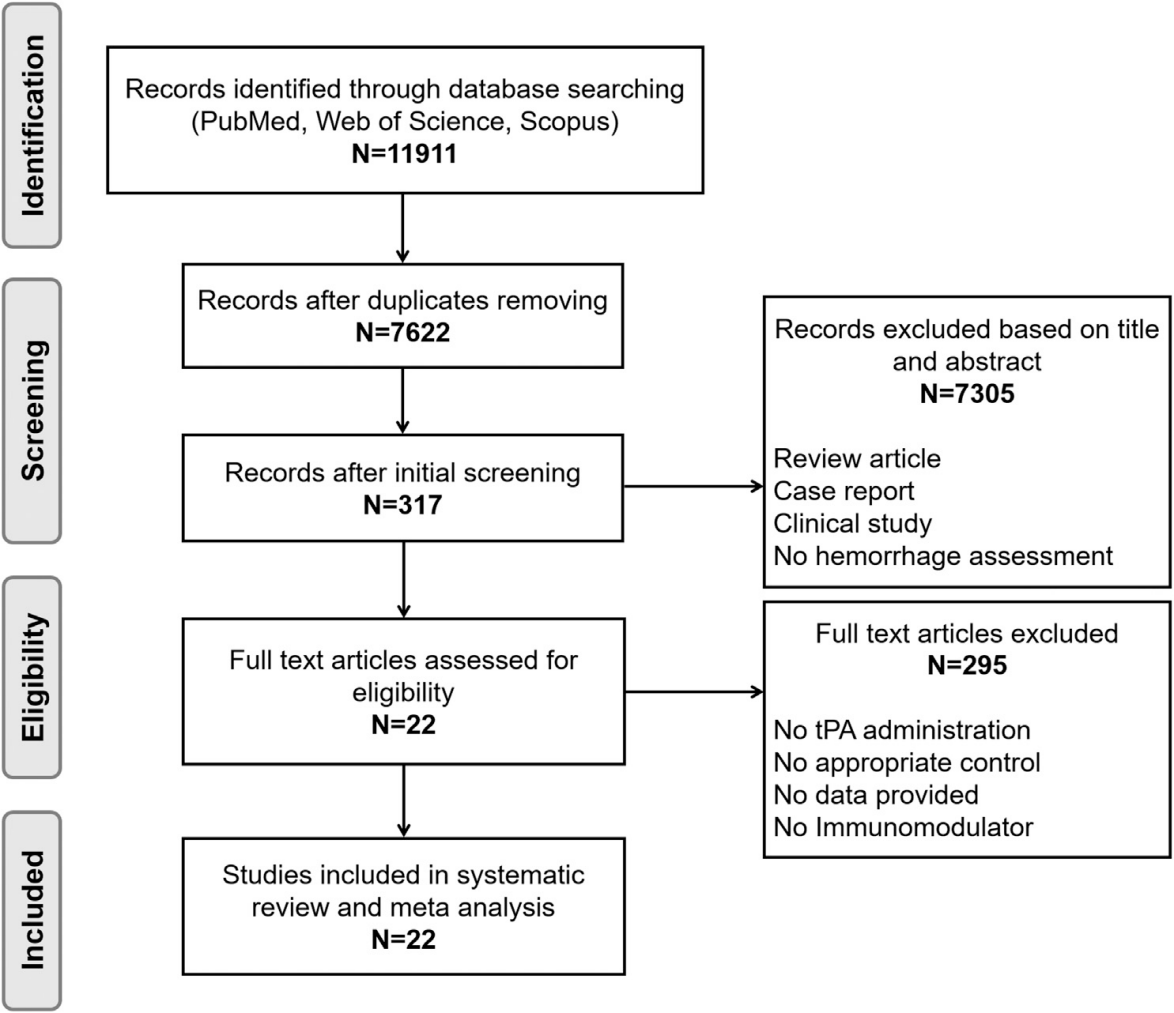
Flow diagram of the study selection process.

Our initial search identified 11,911 publications of which 11,889 were excluded, leaving 22 for inclusion in this systematic review and meta-analysis. The review process is detailed in the flow diagram shown in Figure 1. The 22 included publications described 42 different comparisons for intracerebral hemorrhage, 22 comparisons for infarct size, and 22 comparisons for neurobehavioral outcome. Study characteristics of the included publications are listed in Supplementary Table S1.

### Study Quality

**FIGURE 2:**
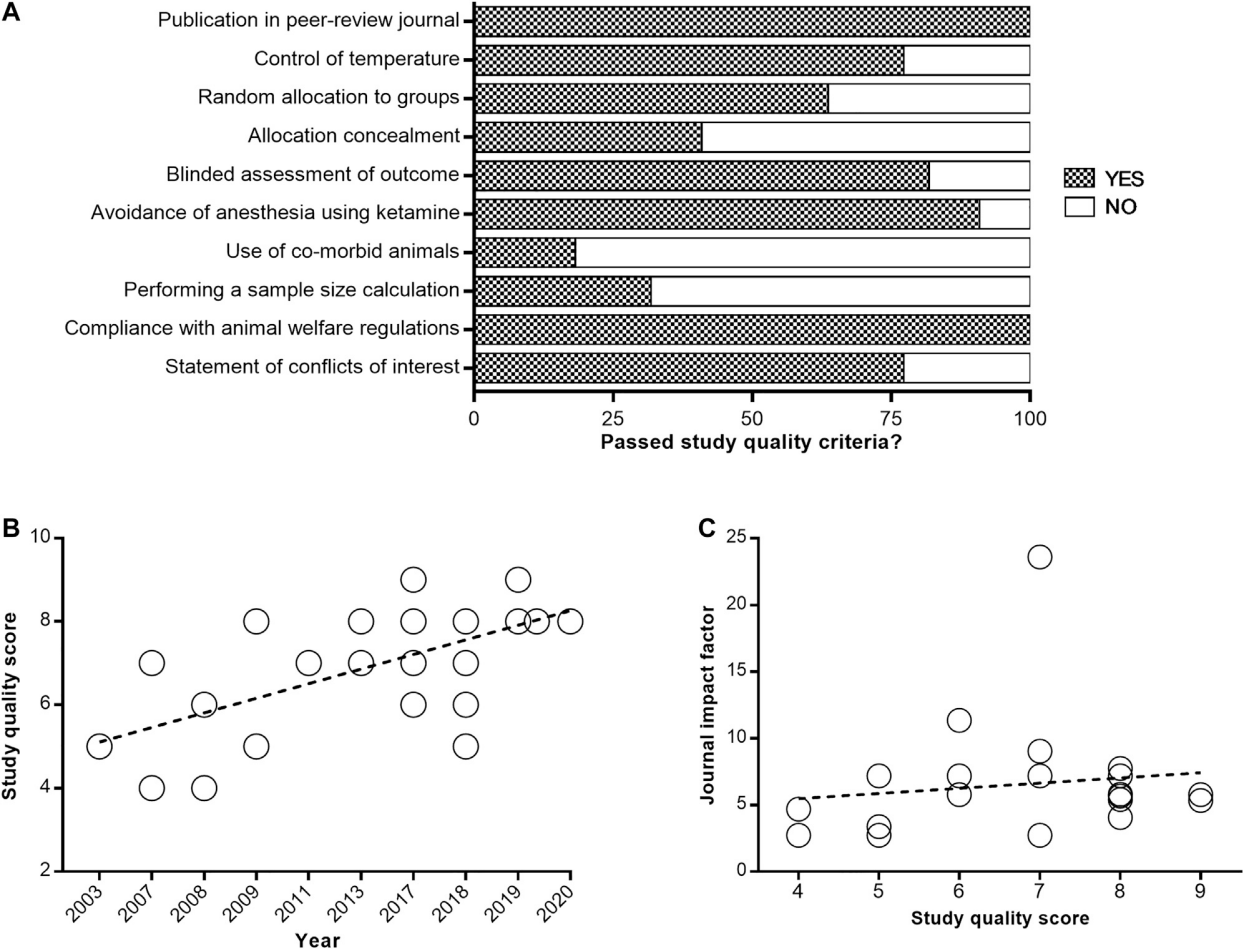
Quality assessment of enrolled studies. Study quality was assessed using the CAMARADES checklist **(A)**. Values are expressed as the percentage of studies reporting each quality indicator. The correlation of study quality with year of publication **(B)** and journal impact factor **(C)**.

The median reported study quality score was 7 of a possible 10 (interquartile range, 6–8) for the 22 included papers. All articles were published in peer-reviewed journals (Figure 2A). Control of temperature during surgery was documented in 17 of 22 papers (77.3%), and random allocation to groups was described in 14 of 22 papers (63.6%). Allocation concealment was reported in 9 of 22 papers (40.9%), whereas blinded assessment was documented in 18 of 22 papers (81.8%). Anesthesia without using ketamine during surgery was reported in 20 of 22 papers (90.9%), whereas use of co-morbid animals was only described in 4 of 22 studies (18.2%). Performed a sample size calculation was documented in 7 of 22 papers (31.8%), and statement of conflicts of interest was reported in 17 of 22 papers (77.3%). All studies reported compliance with animal welfare regulations. A significant correlation between study quality and year of publication was observed, with fresher papers giving higher quality (*R* ^2^ = 77.82%; *p* = 0.0007; Figure 2B). However, no significant correlation was found between study quality and journal impact factor (*R* ^2^ = 1.76%; *p* = 0.5564; Figure 2C). The complete study quality score report is included in Supplemental Material.

### Meta-Analysis

**FIGURE 3:**
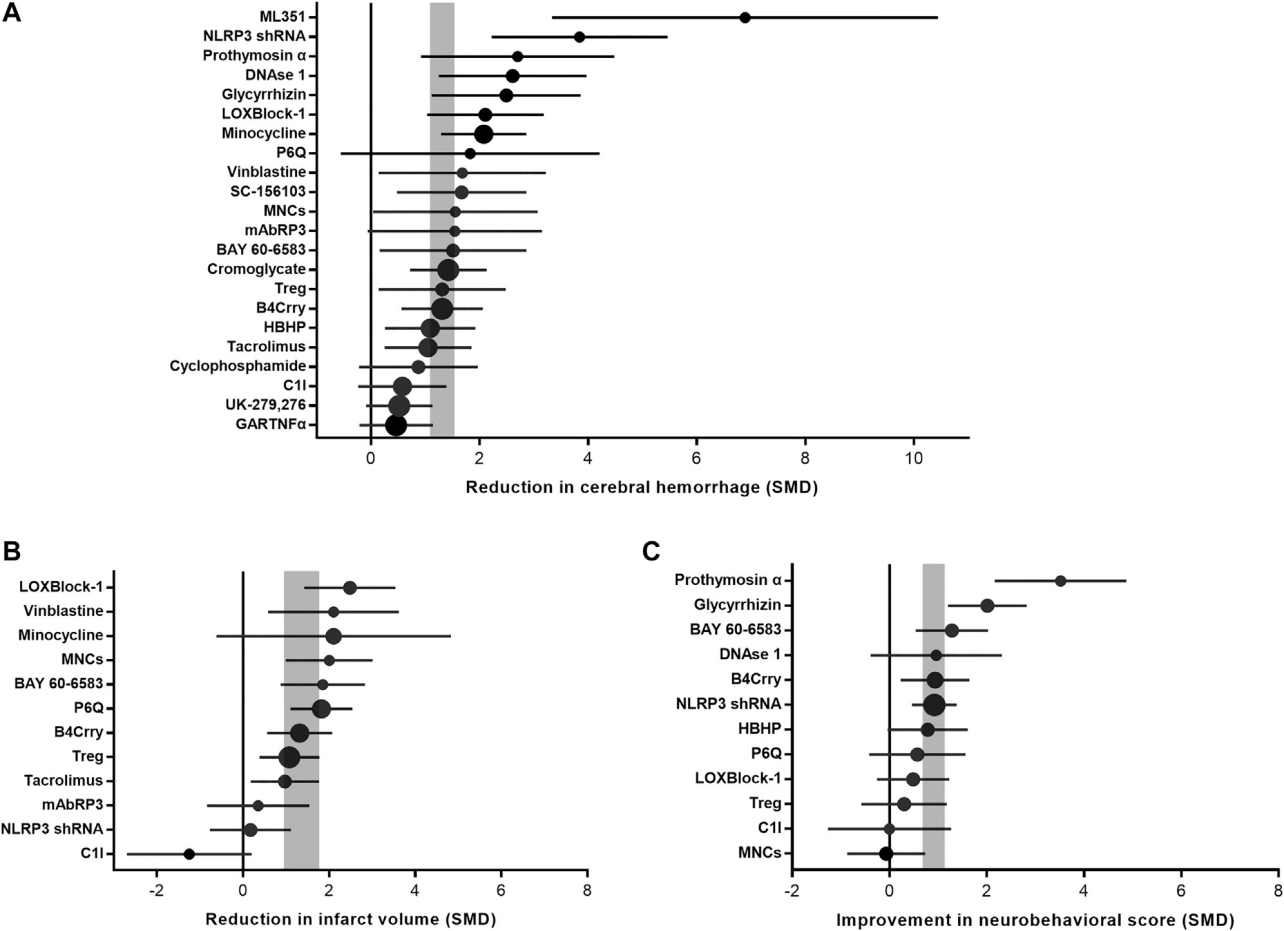
Efficacy of immunomodulators on intracerebral hemorrhage, infarct size, and neurobehavioral score. Forest plots of the effect size in intracerebral hemorrhage **(A)**, infarct size **(B)**, and neurobehavioral score **(C)** calculated using standardized mean differences. Symbol sizes represent the relative number of animals tested for each intervention. The horizontal error bars represent the 95% confidence interval of individual studies. The vertical gray bars represent the 95% confidence interval of the pooled estimate of efficacy.

Intracerebral hemorrhage after immunomodulator administration was improved by 1.31 SMD (95% CI, 1.09–1.52; 42 comparisons; 516 animals), with moderate heterogeneity between studies (*χ* ^2^ = 61.21; *I* ^2^ = 33%; df = 41; *p* = 0.02; Figure 3A; Supplementary Figure S1). Infarct size after immunomodulator administration was improved by 1.35 SMD (95% CI, 0.95–1.76; 22 comparisons; 332 animals), with large heterogeneity between studies (*χ* ^2^ = 46.96; *I* ^2^ = 55%; df = 21; *p* = 0.001; Figure 3B; Supplementary Figure S2). Neurobehavioral outcome after immunomodulator administration was improved by 0.9 SMD (95% CI, 0.67–1.13; 22 comparisons; 380 animals), with moderate heterogeneity between studies (*χ* ^2^ = 41.28; *I* ^2^ = 49%; df = 21; *p* = 0.005; Figure 3C; Supplementary Figure S3).

### Meta-Regression and Subgroup Analysis

**FIGURE 4:**
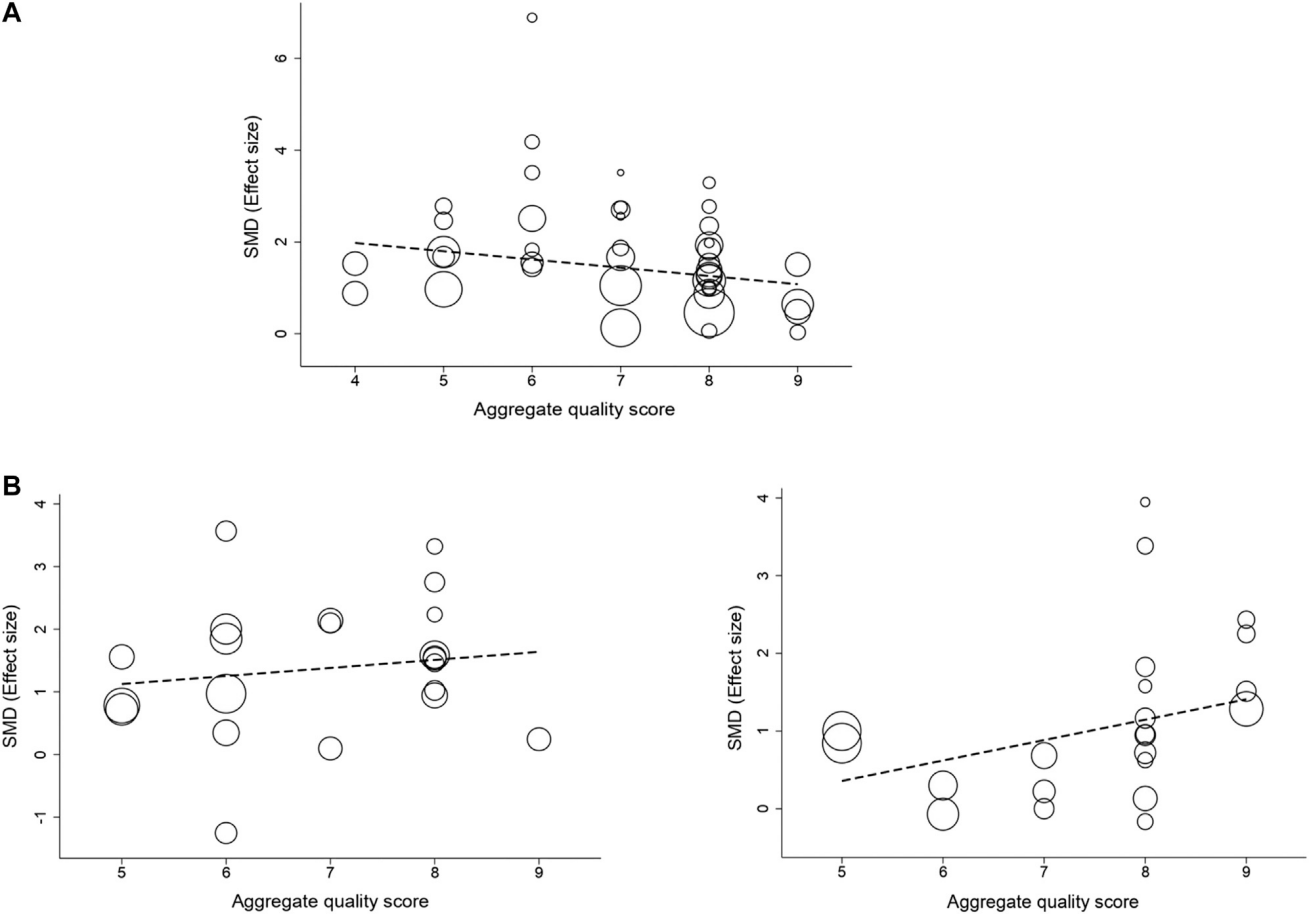
The correlation between study quality score and effect size in intracerebral hemorrhage **(A)**, infarct size **(B)**, and neurobehavioral score **(C)**. The sizes of hollow circles represent the relative number of animals tested for each intervention.

Meta-regression and subgroup analysis were performed to explore the source of heterogeneity. For studies that measured intracerebral hemorrhage, analysis demonstrated that effect size was significantly greater when the study quality score was lower (adjusted *R* ^*2*^ = 13.23%; *p* = 0.01; Figure 4A). For studies that measured infarct size, there was no significant correlation between study quality and effect size (adjusted *R* ^2^ = −6.59%; *p* = 0.14; Figure 4B). However, for studies that measured neurobehavioral outcome, that effect size was significantly greater when the study quality score was higher (adjusted *R* ^2^ = 22.03%; *p* = 0.002; Figure 4C).

**FIGURE 5:**
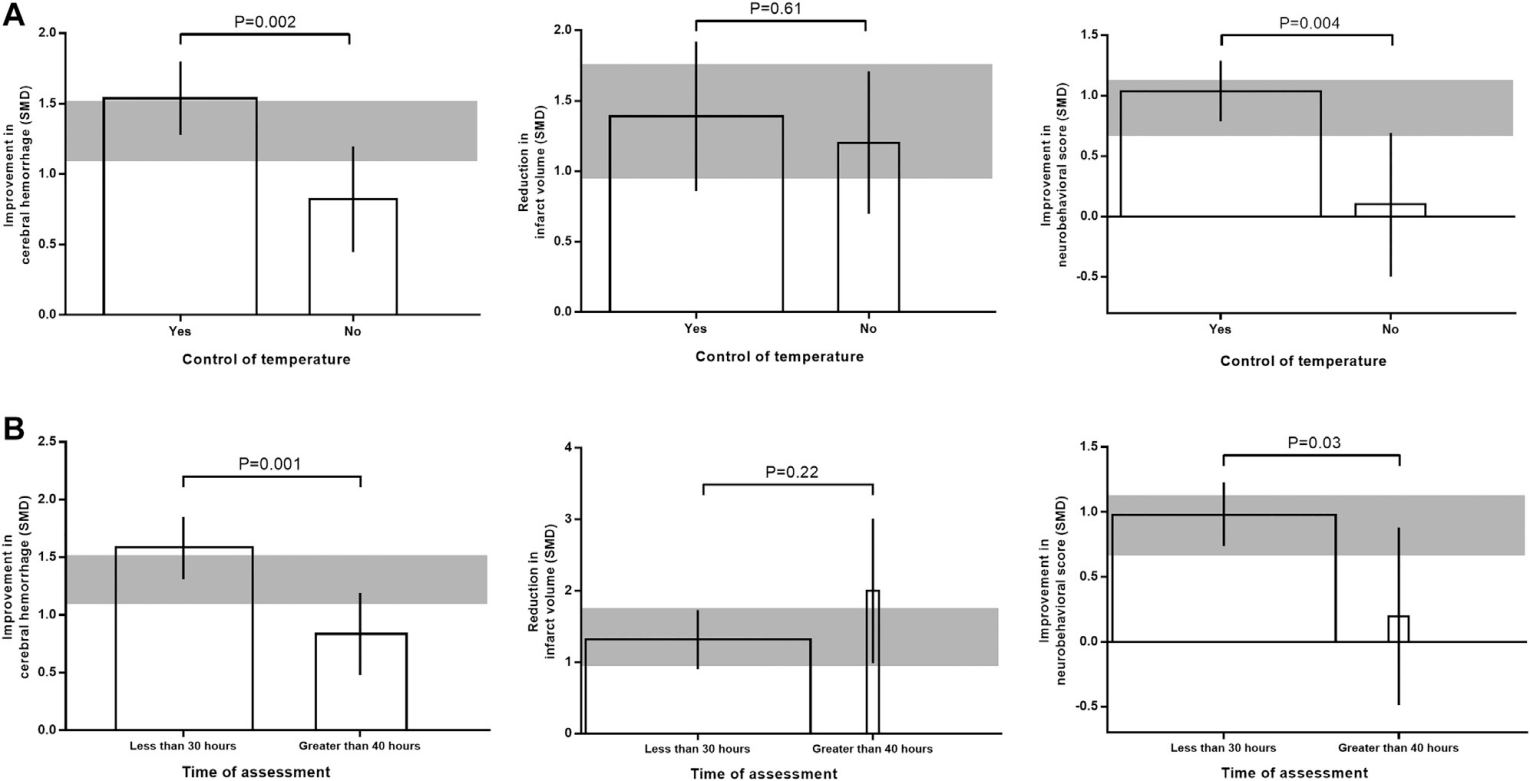
Effects of temperature control **(A)** and time of outcome assessment **(B)** on the efficacy of immunomodulators in intracerebral hemorrhage, infarct size, and neurobehavioral score. The width of each bar represent the relative number of animals in that subgroup. The vertical error bars represent the 95% confidence interval for the individual estimates and the horizontal gray bars represent the 95% confidence interval of the pooled estimate of efficacy.

For intracerebral hemorrhage and neurobehavioral outcome, efficacy were lower in studies that reported control of temperature during surgery (adjusted *R* ^2^ = 1.81%; *p* = 0.002; adjusted *R* ^2^ = 65.95%; *p* = 0.004; Figure 5A). Whereas for infarct size, there was no significant correlation between temperature control and effect size (adjusted *R* ^2^ = −12.52%; *p* = 0.61). For studies that measured intracerebral hemorrhage and neurobehavioral outcome, effect size was significantly greater when outcome was assessed within 30 h after stroke onset (adjusted *R* ^2^ = −8.78%; *p* = 0.001; adjusted *R* ^2^ = 20.44%; *p* = 0.03; Figure 5B). But for studies that measured infarct size, no significant correlation was found between evaluation time and effect size (adjusted *R* ^2^ = 0.82%; *p* = 0.22).

For intracerebral hemorrhage, effect size was significantly greater when studies used chloral hydrate and pentobarbital as anesthetic (adjusted *R* ^2^ = −11.49%; *p* = 0.004; Supplementary Figure S4A). While for infarct size and neurobehavioral outcome, effect size was not significantly changed by anesthetic used (adjusted *R* ^2^ = −48.85%; *p* = 0.22; adjusted *R* ^2^ = −74.01%; *p* = 0.75). For intracerebral hemorrhage and neurobehavioral outcome, no significant correlation was found between route of drug delivery and effect size (adjusted *R* ^2^ = −4.66%; *p* = 0.25; adjusted *R* ^2^ = −30.08%; *p* = 0.31; Supplementary Figure S4B). But for infarct size, the route of drug delivery had an effect on effect size (adjusted *R* ^2^ = −40.77%; *p* = 0.0002). For all the three outcomes, neither blinded assessment nor random allocation contributed significantly to the effect size (Supplementary Figure S5).

### Publication Bias

**FIGURE 6:**
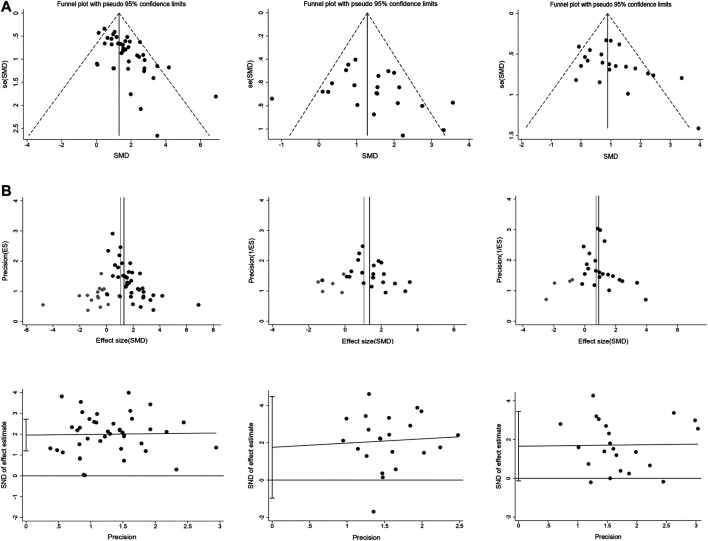
Publication bias assessment. Funnel plots **(A)** for intracerebral hemorrhage, infarct size, and neurobehavioral score showing the publication bias. Trim and fill analysis **(B)** showed the distribution of published study outcomes (black circles) and imputed outcomes (gray circles) in intracerebral hemorrhage, infarct size, and neurobehavioral score. The vertical black lines represent the actual estimate and the gray vertical lines represent the theoretical estimate when publication bias does not exist. Egger’s regression **(C)** for intracerebral hemorrhage, infarct size, and neurobehavioral score confirming potential evidence for publication bias. The short vertical lines represent the 95% confidence interval.

Potential publication bias was assessed by funnel plots, trim and fill method, and Egger’s test. Funnel plots showed obvious asymmetry for intracerebral hemorrhage, and minor asymmetry for infarct size and neurobehavioral outcome (Figure 6A). Trim and fill analysis suggested 14 theoretically missing studies with an adjusted reduction in intracerebral hemorrhage of 1.06 SMD (95% CI, 0.86 to 1.26; compared with 1.31 SMD [95% CI, 1.09–1.52]; Figure 6B). We also estimate five theoretically missing studies with an adjusted reduction in infarct size of 1.04 SMD (95% CI, 0.62 to 1.47; compared with 1.35 SMD [95% CI, 0.95–1.76]), and four unpublished studies with an adjusted improvement in neurobehavioral outcome of 0.74 SMD (95% CI, 0.52 to 0.96; compared with 0.9 SMD [95% CI, 0.67–1.13]). Egger’s regression test indicated significant publication bias for intracerebral hemorrhage (*p* < 0.001; Figure 6C). Whereas Egger's regression showed no publication bias for infarct size (*p* = 0.194) and neurobehavioral outcome (*p* = 0.068).

### Sensitivity Analysis

**FIGURE 7:**
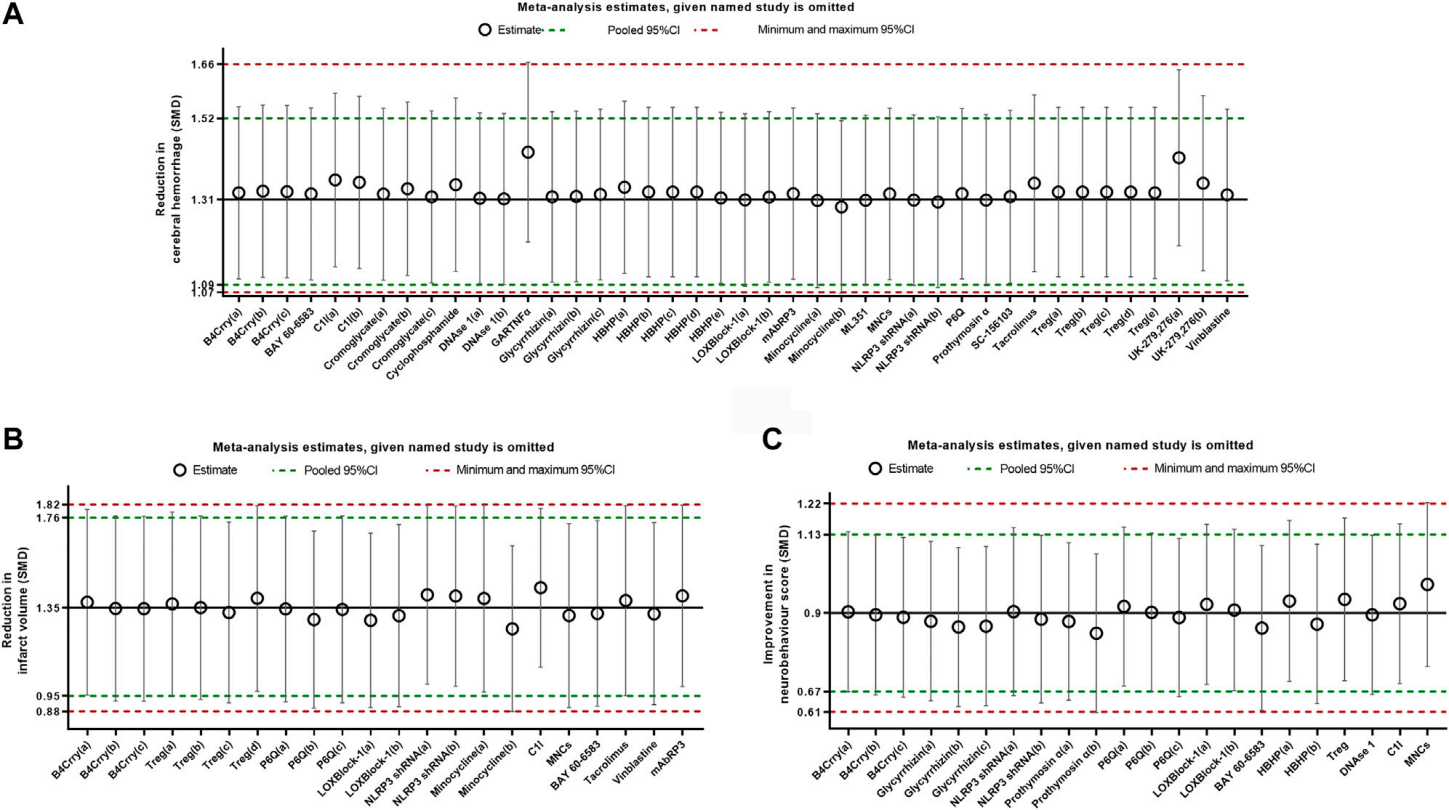
Sensitivity analysis for intracerebral hemorrhage **(A)**, infarct size **(B)**, and neurobehavioral score **(C)** evaluating the robustness of the results. The vertical error bars represent the 95% confidence interval for the individual estimates. The horizontal red bars represent the potentially minimum and maximum 95% confidence interval and the horizontal green bars represent the actual 95% confidence interval of the pooled estimate of efficacy.

Sensitivity analysis was conducted by removing one study at a time to explore whether the results were robust. Results from sensitivity analysis showed that excluding any one study did not affect the results, which demonstrated the stability of our results (Figure 7).

## Discussion

This study evaluated the preclinical literature reporting administration of immunomodulators for the treatment of tPA-induced HT after ischemic stroke. Twenty-two different studies were finally included in this systematic review and meta-analysis. Our results found that immunomodulators led to a reduction in intracerebral hemorrhage, infarct size, and neurobehavioral outcome in animal models of tPA-induced HT. We also found that study quality, temperature control, and evaluation time of outcome were significant factors affecting the efficacy of immunomodulators.

Inflammation plays a critical role in the BBB damage after ischemic stroke(Li et al., 2018). Administration with tPA after stroke exacerbates inflammatory response through various mechanisms including enhancing leukocyte infiltration (Jin et al., 2019) and activating matrix metalloproteinases (MMPs)(Mao et al., 2017). The integrity of BBB was further damaged after tPA treatment, which would eventually lead to lethal intracerebral hemorrhage (Li et al., 2019). Therefore, limiting inflammatory responses may help to reduce the risk of brain hemorrhage and improve the safety of tPA treatment following stroke. Various of immunomodulators have been used in preclinical studies to reduce the risk of hemorrhage induced by tPA treatment. In our analysis, immunomodulators exhibited robust efficacy on tPA-induced intracerebral hemorrhage, cerebral infarction, and neurobehavioral impairments in experimental stroke. Immunomodulators had shown great clinical potential to alleviate cerebral hemorrhage associated with tPA, though relevant clinical trial is lacking.

The study quality overall was high. Only two study received a relatively low score of 4. Some study quality items such as the use of co-morbid animals and sample size calculation were rarely reported. A significant positive correlation was found between study quality and publication year, which was consistent with the previous finding that study quality improved over time (Vahidy et al., 2016). Moreover, there was no significant correlation between study quality and journal impact factor. Paper published in high impact factor journal does not mean it’s of high study quality. This was probably because that even high impact factor journals were mostly low-quality in the early days, but even low impact factor journals were mostly high-quality now. Besides, we also found that study quality is one of the important factors that affect the efficacy of immunomodulators. For intracerebral hemorrhage, a significant negative correlation was found between study quality and effect size. Positive outcomes are more likely to happen in low-quality studies, which is consistent with previous findings (Antonic et al., 2013). However, this theory was inapplicable for infarct size and neurobehavioral outcome in our study. Maybe the relative limited sample size can partly account for the results.

Studies that conducted temperature control during surgery was significantly associated with a higher effect size. This may partly because that animals can get better rehabilitation from good operation environment (Xiao et al., 2013). Furthermore, control of temperature was also highly recommend from the perspective of animal ethics. A significant correlation was also observed between time of assessment and effect size. Larger improvements were seen in studies that reported assessment time less than 30 h after stroke onset. This was possibly because that tPA induced pathological damage was still deteriorating after 30 h from the initiation of stroke.

Our analysis showed that there was no significant correlation of effect size with the anesthetic used, route of drug delivery, blinded assessment, and random allocation (see Supplemental Material). The effects of sample size calculation, animal model used, time of drug delivery, and animal species were also analyzed (data not shown). They had no significant correlation with the efficacy of immunomodulators.

Evidence from funnel plots and Egger’s test showed that obvious asymmetry was observed for intracerebral hemorrhage but only minor asymmetry was found for infarct size and neurobehavioral outcome. After a correction for potential publication bias by using the trim and fill method, the main results for all studies combined were still significant. This suggested that the publication bias observed did not significantly impact this analysis. Sensitivity analysis confirmed that the results of this study were stable.

Although the results of this meta-analysis are very good, the conclusions should be interpreted cautiously given this analysis is based on animal studies. Animal models cannot realistically simulate the pathophysiology involved in patients. Moreover, murine models have markedly different immune systems from humans. So it’s hard to translate the results of animal studies into the clinical setting efficiently, conclusions from the present study also need to be treated with caution.

There are several limitations of this study. First, immunomodulator is a general term for a large class of drugs. It’s hard to evaluate the efficacy of specific class of immunomodulators due to the lack of sufficient studies. Second, no female animals were used in all the included studies, so it is impossible to assess the efficacy of immunomodulators on female animals. Moreover, only English-language publications were included in this study, which may cause publication bias to some degree.

## Conclusion

To the best of our knowledge, this is the first systematic review and meta-analysis which has evaluated the efficacy of immunomodulators on tPA-induced HT in animal models. This meta-analysis confirmed that immunomodulators may improve intracerebral hemorrhage, infarct size, and neurobehavioral outcome in animal models of tPA-induced HT. Furthermore, this study also demonstrated some factors such as study quality score, control of temperature during surgery, and evaluation time of outcome may affect the efficacy of immunomodulators. The results of this study will be help to refine animal experiments in this field and hence reduce the number of animals used in experiments.

## Data Availability Statement

The original contributions presented in the study are included in the article/Supplemental Material, further inquiries can be directed to the corresponding authors.

## Author Contributions

Conception and design: YY and H-XT. Screening of titles and abstracts, full-text data extraction: YY and Y-TZ. Analysis and interpretation of data, drafting the article: YY and J-YH. Critically revising the article: J-YH, Y-TZ, and H-XT. Statistical analysis: YY.

## Funding

This study was supported by the National Natural Science Foundation of China (Grant No. 81903942) and the China Postdoctoral Science Foundation (Grant No. 2019M650393).

## Conflict of Interest

The authors declare that the research was conducted in the absence of any commercial or financial relationships that could be construed as a potential conflict of interest.
