# Supplementary material for: The Protective Role of Immunomodulators on Tissue-Type Plasminogen Activator-Induced Hemorrhagic Transformation in Experimental Stroke: A Systematic Review and Meta-Analysis
Source: Front Pharmacol. 2020 Dec 15;11:615166. doi: 10.3389/fphar.2020.615166 (PMC7793743; doi:10.3389/fphar.2020.615166)
Supplement: Supplementary file 1 [file datasheet1.pdf]

Supplementary Material

Efficacy of Immunomodulators on Tissue-Type Plasminogen Activator-Induced Hemorrhagic Transformation in Experimental Stroke: A Systematic Review and Meta-Analysis

Yang Ye[1,2†]*, Yu-Tian Zhu[3,4†] and Hong-Xuan Tong[5]*

[1] *Department of Integration of Chinese and Western Medicine, School of Basic Medical Sciences, Peking University, Beijing, China,*

[2] *Tasly Microcirculation Research Center, Peking University Health Science Center, Beijing, China,*

[3] *Department of Traditional Chinese Medicine, Peking University Third Hospital, Beijing, China,*

[4] *Department of Urology, Peking University Third Hospital, Beijing, China,*

[5] *Institute of Basic Theory for Chinese Medicine, China Academy of Chinese Medical Sciences, Beijing, China*

***Correspondence:**

Yang Ye, yeyang89@126.com

Hong-Xuan Tong, thongxuan1@163.com

[†]These authors have contributed equally to this work and share first authorship

Supplementary I: Search strategy

1. Search strategy used in PubMed database:

#1. tPA OR rtPA OR t-PA OR rt-PA OR tissue plasminogen activator OR tissue-plasminogen activator OR alteplase[Title/Abstract]

#2. hemorrhagic transformation OR hemorrhage OR haemorrhage OR bleeding[Title/Abstract]

#3. stroke OR ischemia OR cerebral OR brain[Title/Abstract]

#4. #1 AND #2 AND #3

Items found: 2,991 (By July 26, 2020)

2. Search strategy used in Web of Science database:

#1. TS=(tPA OR rtPA OR t-PA OR rt-PA OR tissue plasminogen activator OR tissue-plasminogen activator OR alteplase)

#2. TS=(hemorrhagic transformation OR hemorrhage OR haemorrhage OR bleeding)

#3. TS=(stroke OR ischemia OR cerebral OR brain)

#4. #1 AND #2 AND #3

Items found: 7655 (By July 26, 2020)

3. Search strategy used in Scopus database:

#1. TITLE-ABS-KEY(tPA OR rtPA OR t-PA OR rt-PA OR tissue plasminogen activator OR tissue-plasminogen activator OR alteplase)

#2. TITLE-ABS-KEY(hemorrhagic transformation OR hemorrhage OR haemorrhage OR bleeding)

#3. TITLE-ABS-KEY(stroke OR ischemia OR cerebral OR brain)

#4. #1 AND #2 AND #3

Items found: 1265 (By July 26, 2020)

Supplementary II: Figures and Tables

Study or Subgroup	tPA Mean	tPA SD	tPA Total	Immunomodulator Mean	Immunomodulator SD	Immunomodulator Total	Weight	Std. Mean Difference IV, Fixed, 95% CI
**1.1.1 B4Crry**								
B4Crry(a)	0.35	0.17	6	0.14	0.1	6	2.6%	1.39 [0.07, 2.71]
B4Crry(b)	0.57	0.24	6	0.33	0.07	6	2.8%	1.25 [-0.03, 2.54]
B4Crry(c)	0.75	0.2	6	0.47	0.2	6	2.7%	1.29 [-0.00, 2.59]
**Subtotal (95% CI)**			**18**			**18**	**8.2%**	**1.31 [0.56, 2.06]**
Heterogeneity: Chi^2 = 0.02, df = 2 (P = 0.99); I^2 = 0%								
Test for overall effect: Z = 3.42 (P = 0.0006)								
**1.1.2 Glycyrrhizin**								
Glycyrrhizin(a)	30.65	5.63	2	20.17	1.69	6	0.6%	3.29 [0.52, 6.06]
Glycyrrhizin(b)	30.65	5.63	2	16.34	4.28	6	0.8%	2.75 [0.27, 5.22]
Glycyrrhizin(c)	30.65	5.63	2	16.68	6.65	6	1.1%	1.87 [-0.18, 3.93]
**Subtotal (95% CI)**			**6**			**18**	**2.4%**	**2.49 [1.12, 3.86]**
Heterogeneity: Chi^2 = 0.71, df = 2 (P = 0.70); I^2 = 0%								
Test for overall effect: Z = 3.55 (P = 0.0004)								
**1.1.3 HBHP**								
HBHP(a)	75.89	4.91	5	65.18	14.73	5	2.6%	0.88 [-0.45, 2.22]
HBHP(b)	117.86	17.14	1	116.79	28.21	5	1.0%	0.03 [-2.12, 2.18]
HBHP(c)	117.86	17.14	1	82.5	28.93	5	0.8%	0.98 [-1.37, 3.32]
HBHP(d)	117.86	17.14	1	90.71	21.43	5	0.8%	1.01 [-1.35, 3.37]
HBHP(e)	146.99	25.3	5	88.55	19.28	5	1.4%	2.35 [0.54, 4.16]
**Subtotal (95% CI)**			**13**			**25**	**6.6%**	**1.09 [0.26, 1.92]**
Heterogeneity: Chi^2 = 2.89, df = 4 (P = 0.58); I^2 = 0%								
Test for overall effect: Z = 2.57 (P = 0.01)								
**1.1.4 Treg**								
Treg(a)	14.48	2.15	1	14.01	5.38	4	1.0%	0.06 [-2.13, 2.26]
Treg(b)	14.48	2.15	1	9.36	1.88	4	0.4%	1.98 [-1.47, 5.43]
Treg(c)	14.48	2.15	1	5.99	1.76	4	0.2%	3.51 [-1.70, 8.71]
Treg(d)	14.48	2.15	1	4.98	2.7	4	0.3%	2.56 [-1.52, 6.64]
Treg(e)	11.07	4.96	4	3.56	4	4	1.6%	1.45 [-0.26, 3.16]
**Subtotal (95% CI)**			**8**			**20**	**3.4%**	**1.31 [0.14, 2.48]**
Heterogeneity: Chi^2 = 2.46, df = 4 (P = 0.65); I^2 = 0%								
Test for overall effect: Z = 2.20 (P = 0.03)								
**1.1.5 Cromoglycate**								
Cromoglycate(a)	0.36	0.29	6	0.02	0.02	6	2.5%	1.53 [0.17, 2.88]
Cromoglycate(b)	0.3	0.24	9	0.08	0.09	9	4.4%	1.16 [0.14, 2.17]
Cromoglycate(c)	0.34	0.22	6	0.02	0.05	6	2.2%	1.85 [0.40, 3.30]
**Subtotal (95% CI)**			**21**			**21**	**9.1%**	**1.42 [0.72, 2.13]**
Heterogeneity: Chi^2 = 0.62, df = 2 (P = 0.73); I^2 = 0%								
Test for overall effect: Z = 3.94 (P < 0.0001)								
**1.1.6 NLRP3 shRNA**								
NLRP3 shRNA(a)	18.12	4.31	3	4.71	3.22	8	0.9%	3.51 [1.25, 5.77]
NLRP3 shRNA(b)	18.12	4.31	3	3.6	2.94	10	0.9%	4.18 [1.86, 6.49]
**Subtotal (95% CI)**			**6**			**18**	**1.8%**	**3.84 [2.22, 5.46]**
Heterogeneity: Chi^2 = 0.16, df = 1 (P = 0.69); I^2 = 0%								
Test for overall effect: Z = 4.65 (P < 0.00001)								
**1.1.7 DNAse 1**								
DNAse 1(a)	27.57	5.65	5	13.33	4.75	5	1.3%	2.46 [0.60, 4.32]
DNAse 1(b)	27.34	6.55	5	11.3	3.39	5	1.2%	2.78 [0.78, 4.77]
**Subtotal (95% CI)**			**10**			**10**	**2.5%**	**2.61 [1.25, 3.97]**
Heterogeneity: Chi^2 = 0.05, df = 1 (P = 0.82); I^2 = 0%								
Test for overall effect: Z = 3.76 (P = 0.0002)								
**1.1.8 C1I**								
C1I(a)	38.1	26.19	7	21.1	23.76	8	4.2%	0.64 [-0.41, 1.69]
C1I(b)	2.5	1.47	6	1.8	1	4	2.7%	0.48 [-0.81, 1.78]
**Subtotal (95% CI)**			**13**			**12**	**6.9%**	**0.58 [-0.24, 1.39]**
Heterogeneity: Chi^2 = 0.04, df = 1 (P = 0.85); I^2 = 0%								
Test for overall effect: Z = 1.39 (P = 0.16)								
**1.1.9 UK-279,276**								
UK-279,276(a)	31.2	58.8	11	24.5	42.6	11	6.6%	0.13 [-0.71, 0.96]
UK-279,276(b)	74.8	59.9	11	23.3	40.1	11	5.8%	0.97 [0.08, 1.87]
**Subtotal (95% CI)**			**22**			**22**	**12.3%**	**0.52 [-0.09, 1.13]**
Heterogeneity: Chi^2 = 1.84, df = 1 (P = 0.18); I^2 = 46%								
Test for overall effect: Z = 1.67 (P = 0.09)								

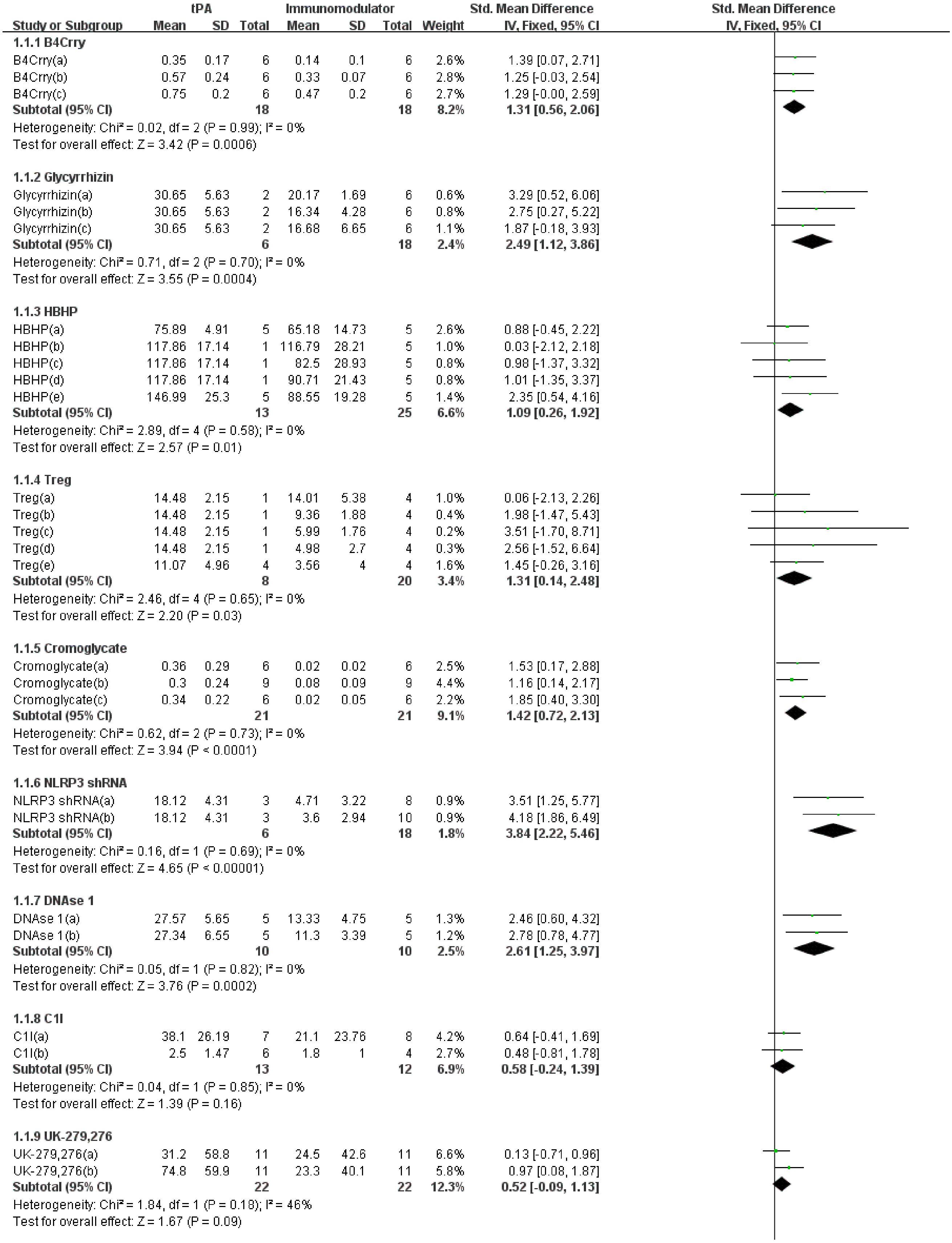

**1.1.10 Minocycline**								
Minocycline(a)	5.33	0.62	11	4.27	0.52	11	4.4%	1.78 [0.76, 2.80]
Minocycline(b)	11.81	3.96	9	3.95	1.92	11	3.0%	2.51 [1.27, 3.74]
**Subtotal (95% CI)**			**20**			**22**	**7.5%**	**2.08 [1.29, 2.86]**
Heterogeneity: Chi² = 0.79, df = 1 (P = 0.38); I² = 0%								
Test for overall effect: Z = 5.18 (P < 0.00001)								
**1.1.11 LOXBlock-1**								
LOXBlock-1(a)	22.4	8.1	9	8.8	4.53	8	3.2%	1.93 [0.73, 3.14]
LOXBlock-1(b)	2.17	0.74	4	0.39	0.28	4	0.8%	2.77 [0.41, 5.12]
**Subtotal (95% CI)**			**13**			**12**	**4.0%**	**2.11 [1.03, 3.18]**
Heterogeneity: Chi² = 0.38, df = 1 (P = 0.54); I² = 0%								
Test for overall effect: Z = 3.84 (P = 0.0001)								
**1.1.12 Others**								
BAY 60-6583	32.76	6.35	6	24.4	3.45	6	2.5%	1.51 [0.16, 2.86]
Cyclophosphamide	0.21	0.14	9	0.09	0.11	6	3.8%	0.87 [-0.22, 1.97]
GARTNFα	2.16	2.47	17	1.08	2.08	18	10.2%	0.46 [-0.21, 1.14]
mAbRP3	10.8	4.68	3	5.2	2.65	7	1.8%	1.54 [-0.06, 3.15]
ML351	9.08	0.31	5	3.51	0.93	7	0.4%	6.89 [3.33, 10.44]
MNCs	3.8	0.84	5	2	1.22	5	2.0%	1.55 [0.04, 3.07]
P6Q	0.6	0.28	3	0.13	0.08	3	0.8%	1.83 [-0.56, 4.21]
Prothymosin α	0.56	0.22	8	0.02	0.03	4	1.4%	2.70 [0.92, 4.48]
SC-156103	10.04	2	7	6.39	2.11	9	3.2%	1.67 [0.48, 2.86]
Tacrolimus	2.31	0.75	14	1.52	0.71	14	7.2%	1.05 [0.25, 1.85]
Vinblastine	10.8	4.68	3	4.6	3	9	1.9%	1.68 [0.14, 3.22]
**Subtotal (95% CI)**			**80**			**88**	**35.3%**	**1.19 [0.82, 1.55]**
Heterogeneity: Chi² = 19.46, df = 10 (P = 0.03); I² = 49%								
Test for overall effect: Z = 6.44 (P < 0.00001)								
**Total (95% CI)**			**230**			**286**	**100.0%**	**1.31 [1.09, 1.52]**
Heterogeneity: Chi² = 61.21, df = 41 (P = 0.02); I² = 33%								
Test for overall effect: Z = 11.96 (P < 0.00001)								
Test for subgroup differences: Chi² = 31.79, df = 11 (P = 0.0008), I² = 65.4%								

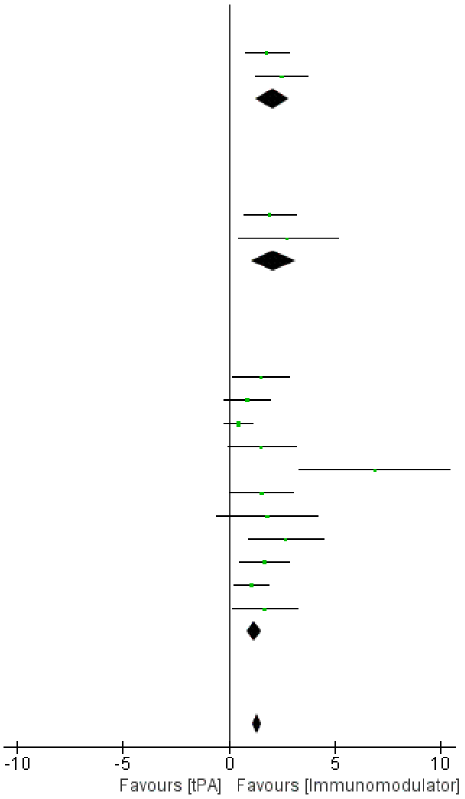

Figure S1. Meta-analysis of immunomodulator on cerebral hemorrhage. The horizontal lines indicate 95% confidence intervals for individual standardized mean differences (SMD), and the black diamonds indicates global SMD and confidence interval.

Study or Subgroup	tPA Mean	tPA SD	tPA Total	Immunomodulator Mean	Immunomodulator SD	Immunomodulator Total	Weight	Std. Mean Difference IV, Random, 95% CI
**1.2.1 B4Crry**								
B4Crry(a)	3.06	1.641158	6	1.72	0.857321	6	4.8%	0.94 [-0.28, 2.17]
B4Crry(b)	7.88	5.339888	6	1.4	1.420704	6	4.4%	1.53 [0.17, 2.89]
B4Crry(c)	10.56	5.119434	6	4.06	1.910602	6	4.3%	1.55 [0.19, 2.91]
**Subtotal (95% CI)**			**18**			**18**	**13.5%**	**1.31 [0.56, 2.07]**
Heterogeneity: Tau^2 = 0.00; Chi^2 = 0.57, df = 2 (P = 0.75); I^2 = 0%								
Test for overall effect: Z = 3.41 (P = 0.0007)								
**1.2.2 Treg**								
Treg(a)	59.54	4.6	4	53.56	5.52	4	3.8%	1.02 [-0.53, 2.58]
Treg(b)	63.17	13.84	4	44.5	7.38	4	3.4%	1.46 [-0.25, 3.18]
Treg(c)	54.11	4.66	4	35.92	8.86	4	2.6%	2.23 [0.17, 4.30]
Treg(d)	49.53	14.93	10	36.17	20.56267	8	5.8%	0.72 [-0.25, 1.69]
**Subtotal (95% CI)**			**22**			**20**	**15.5%**	**1.08 [0.38, 1.77]**
Heterogeneity: Tau^2 = 0.00; Chi^2 = 1.92, df = 3 (P = 0.59); I^2 = 0%								
Test for overall effect: Z = 3.03 (P = 0.002)								
**1.2.3 P6Q**								
P6Q(a)	35.65	5.5	5	25.19	6.65	10	4.7%	1.56 [0.30, 2.81]
P6Q(b)	35.65	5.5	5	17.61	6.45	10	3.7%	2.75 [1.18, 4.32]
P6Q(c)	45.9	9.49	9	33.39	5.24	10	5.4%	1.58 [0.52, 2.65]
**Subtotal (95% CI)**			**19**			**30**	**13.8%**	**1.82 [1.10, 2.54]**
Heterogeneity: Tau^2 = 0.00; Chi^2 = 1.71, df = 2 (P = 0.43); I^2 = 0%								
Test for overall effect: Z = 4.95 (P < 0.00001)								
**1.2.4 LOXBlock-1**								
LOXBlock-1(a)	53	14.45	6	12.3	6.86	6	2.8%	3.32 [1.34, 5.30]
LOXBlock-1(b)	48	13.2	9	23.7	7.071068	8	4.7%	2.14 [0.88, 3.40]
**Subtotal (95% CI)**			**15**			**14**	**7.5%**	**2.48 [1.42, 3.54]**
Heterogeneity: Tau^2 = 0.00; Chi^2 = 0.98, df = 1 (P = 0.32); I^2 = 0%								
Test for overall effect: Z = 4.58 (P < 0.00001)								
**1.2.5 NLRP3 shRNA**								
NLRP3 shRNA(a)	41.91	15.32865	3	39.83	20.25154	8	4.5%	0.10 [-1.23, 1.43]
NLRP3 shRNA(b)	41.91	15.32865	3	36.57	20.98693	8	4.4%	0.25 [-1.09, 1.58]
**Subtotal (95% CI)**			**6**			**16**	**8.9%**	**0.17 [-0.77, 1.11]**
Heterogeneity: Tau^2 = 0.00; Chi^2 = 0.02, df = 1 (P = 0.88); I^2 = 0%								
Test for overall effect: Z = 0.36 (P = 0.72)								
**1.2.6 Minocycline**								
Minocycline(a)	37.11	6.33	11	31.13	8.27	11	6.1%	0.78 [-0.09, 1.65]
Minocycline(b)	387.27	21.93	9	215.03	58.87009	11	3.9%	3.57 [2.05, 5.08]
**Subtotal (95% CI)**			**20**			**22**	**10.0%**	**2.10 [-0.62, 4.83]**
Heterogeneity: Tau^2 = 3.48; Chi^2 = 9.74, df = 1 (P = 0.002); I^2 = 90%								
Test for overall effect: Z = 1.51 (P = 0.13)								
**1.2.13 Others**								
BAY 60-6583	49.69	9.04	12	36.33	3.95	12	5.7%	1.85 [0.87, 2.83]
C1I	85	46.54	6	142	30	4	4.1%	-1.25 [-2.70, 0.20]
mAbRP3	243	32	4	221	66	9	4.9%	0.35 [-0.84, 1.54]
MNCs	146	17.2	12	115	12.3	12	5.6%	2.00 [0.99, 3.01]
Tacrolimus	18.29	3.29	14	13.64	5.69	14	6.5%	0.97 [0.18, 1.76]
Vinblastine	243	32	4	159	39	9	3.9%	2.10 [0.58, 3.62]
**Subtotal (95% CI)**			**52**			**60**	**30.7%**	**1.05 [0.17, 1.93]**
Heterogeneity: Tau^2 = 0.85; Chi^2 = 18.66, df = 5 (P = 0.002); I^2 = 73%								
Test for overall effect: Z = 2.35 (P = 0.02)								
**Total (95% CI)**			**152**			**180**	**100.0%**	**1.35 [0.95, 1.76]**
Heterogeneity: Tau^2 = 0.49; Chi^2 = 46.96, df = 21 (P = 0.0010); I^2 = 55%								
Test for overall effect: Z = 6.59 (P < 0.00001)								
Test for subgroup differences: Chi^2 = 13.31, df = 6 (P = 0.04), I^2 = 54.9%								

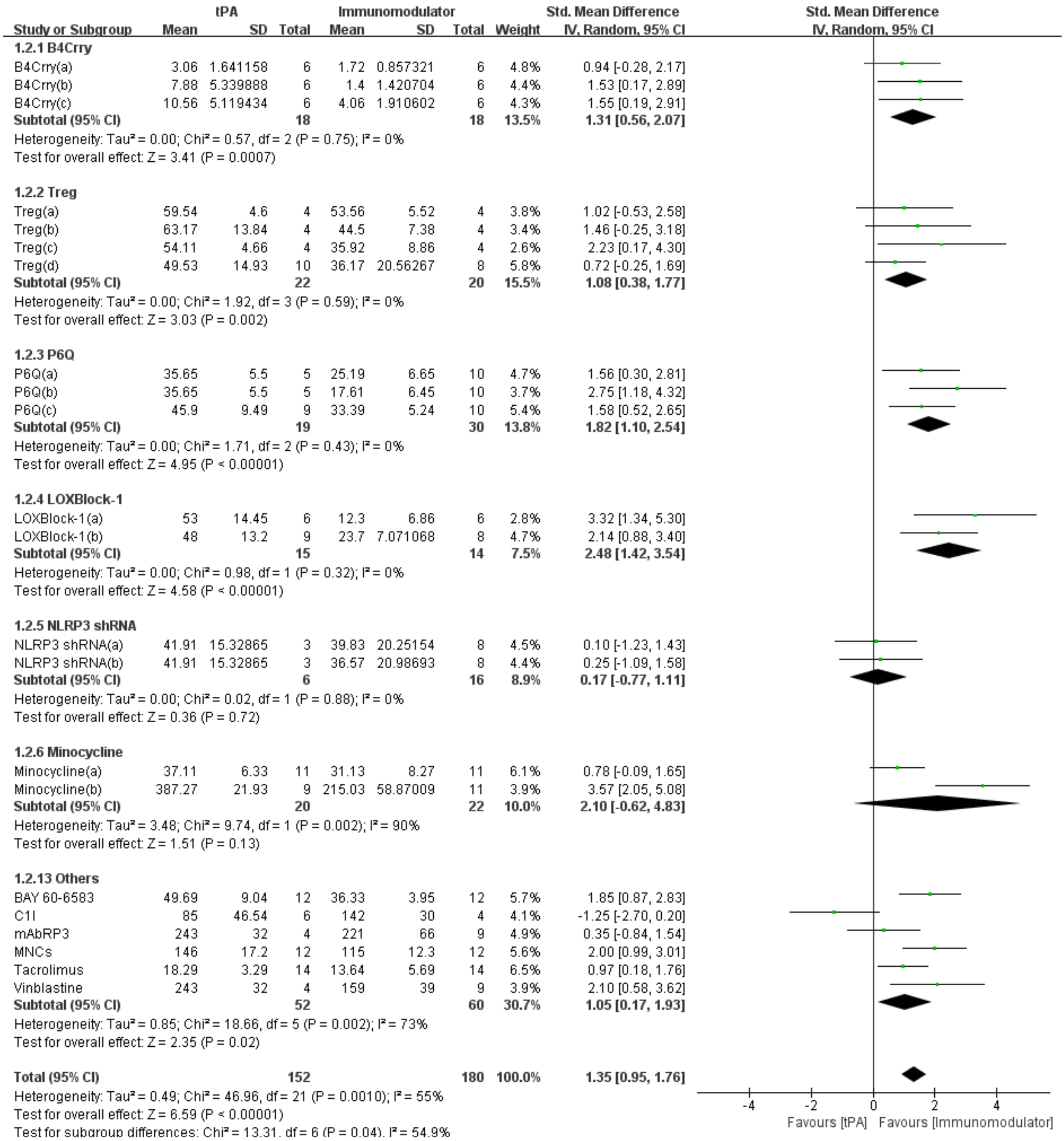

Figure S2. Meta-analysis of immunomodulator on infarct volume. The horizontal lines indicate 95% confidence intervals for individual standardized mean differences (SMD), and the black diamonds indicates global SMD and confidence interval.

	tPA			Immunomodulator				Std. Mean Difference
Study or Subgroup	Mean	SD	Total	Mean	SD	Total	Weight	IV, Fixed, 95% CI
**1.3.1 B4Crry**								
B4Crry(a)	1.37	1.2	6	0.6	0.71	6	3.8%	0.72 [-0.46, 1.91]
B4Crry(b)	2.37	1.05	6	1.36	0.91	6	3.5%	0.95 [-0.27, 2.17]
B4Crry(c)	2.87	1.03	6	1.47	1.18	6	3.3%	1.17 [-0.10, 2.43]
**Subtotal (95% CI)**			**18**			**18**	**10.6%**	**0.94 [0.23, 1.64]**
Heterogeneity: Chi² = 0.25, df = 2 (P = 0.88); I² = 0%								
Test for overall effect: Z = 2.60 (P = 0.009)								
**1.3.2 Glycyrrhizin**								
Glycyrrhizin(a)	10.2	1.53	4	6.02	2.84	12	3.2%	1.51 [0.23, 2.79]
Glycyrrhizin(b)	10.2	1.53	4	6.1	1.61	12	2.4%	2.43 [0.94, 3.92]
Glycyrrhizin(c)	10.2	1.53	4	5.6	2.03	12	2.5%	2.25 [0.80, 3.69]
**Subtotal (95% CI)**			**12**			**36**	**8.1%**	**2.01 [1.20, 2.82]**
Heterogeneity: Chi² = 1.00, df = 2 (P = 0.61); I² = 0%								
Test for overall effect: Z = 4.89 (P < 0.00001)								
**1.3.3 NLRP3 shRNA**								
NLRP3 shRNA(a)	-0.53	0.78	15	-1.53	1.31	30	12.7%	0.84 [0.20, 1.49]
NLRP3 shRNA(b)	-0.53	0.78	15	-1.77	1.38	30	12.3%	1.00 [0.34, 1.66]
**Subtotal (95% CI)**			**30**			**60**	**24.9%**	**0.92 [0.46, 1.38]**
Heterogeneity: Chi² = 0.11, df = 1 (P = 0.74); I² = 0%								
Test for overall effect: Z = 3.92 (P < 0.0001)								
**1.3.4 Prothymosin α**								
Prothymosin α(a)	5.02	0.22	5	4.08	0.2	4	0.7%	3.95 [1.18, 6.71]
Prothymosin α(b)	4.99	0.3	9	1.59	1.32	9	2.2%	3.38 [1.83, 4.94]
**Subtotal (95% CI)**			**14**			**13**	**2.9%**	**3.52 [2.16, 4.87]**
Heterogeneity: Chi² = 0.12, df = 1 (P = 0.73); I² = 0%								
Test for overall effect: Z = 5.09 (P < 0.00001)								
**1.3.5 P6Q**								
P6Q(a)	-118.295	57.23	2	-112.0959	24.49	6	2.1%	-0.17 [-1.77, 1.44]
P6Q(b)	-118.295	57.23	2	-149.478	39.96	6	1.9%	0.63 [-1.03, 2.28]
P6Q(c)	-118.295	57.23	2	-182.4493	29.13	6	1.4%	1.58 [-0.36, 3.51]
**Subtotal (95% CI)**			**6**			**18**	**5.4%**	**0.57 [-0.42, 1.56]**
Heterogeneity: Chi² = 1.85, df = 2 (P = 0.40); I² = 0%								
Test for overall effect: Z = 1.14 (P = 0.26)								
**1.3.6 LOXBlock-1**								
LOXBlock-1(a)	3.22	3.53	6	2.49	2.38	6	4.1%	0.22 [-0.91, 1.36]
LOXBlock-1(b)	4.59	2.43	9	2.83	2.46	8	5.4%	0.68 [-0.30, 1.67]
**Subtotal (95% CI)**			**15**			**14**	**9.5%**	**0.49 [-0.26, 1.23]**
Heterogeneity: Chi² = 0.36, df = 1 (P = 0.55); I² = 0%								
Test for overall effect: Z = 1.28 (P = 0.20)								
**1.3.7 HBHP**								
HBHP(a)	5.59	1.19	7	5.4	1.49	7	4.8%	0.13 [-0.92, 1.18]
HBHP(b)	9.05	0.96	6	6.22	1.72	8	3.0%	1.82 [0.50, 3.15]
**Subtotal (95% CI)**			**13**			**15**	**7.8%**	**0.78 [-0.04, 1.61]**
Heterogeneity: Chi² = 3.85, df = 1 (P = 0.05); I² = 74%								
Test for overall effect: Z = 1.87 (P = 0.06)								
**1.3.8 Others**								
BAY 60-6583	3.82	0.95	17	2.53	1.01	17	9.5%	1.28 [0.54, 2.03]
C1I	2.5	0.73	6	2.5	0.6	4	3.3%	0.00 [-1.27, 1.27]
DNAse 1	73.94	10.68	5	63.42	9.11	5	2.9%	0.96 [-0.39, 2.31]
MNCs	3.53	0.61	12	3.58	0.74	12	8.3%	-0.07 [-0.87, 0.73]
Treg	62.92	18.02	10	56.93	20.3	10	6.8%	0.30 [-0.58, 1.18]
**Subtotal (95% CI)**			**50**			**48**	**30.7%**	**0.53 [0.12, 0.95]**
Heterogeneity: Chi² = 7.41, df = 4 (P = 0.12); I² = 46%								
Test for overall effect: Z = 2.52 (P = 0.01)								
**Total (95% CI)**			**158**			**222**	**100.0%**	**0.90 [0.67, 1.13]**
Heterogeneity: Chi² = 41.28, df = 21 (P = 0.005); I² = 49%								
Test for overall effect: Z = 7.64 (P < 0.00001)								
Test for subgroup differences: Chi² = 26.32, df = 7 (P = 0.0004), I² = 73.4%								

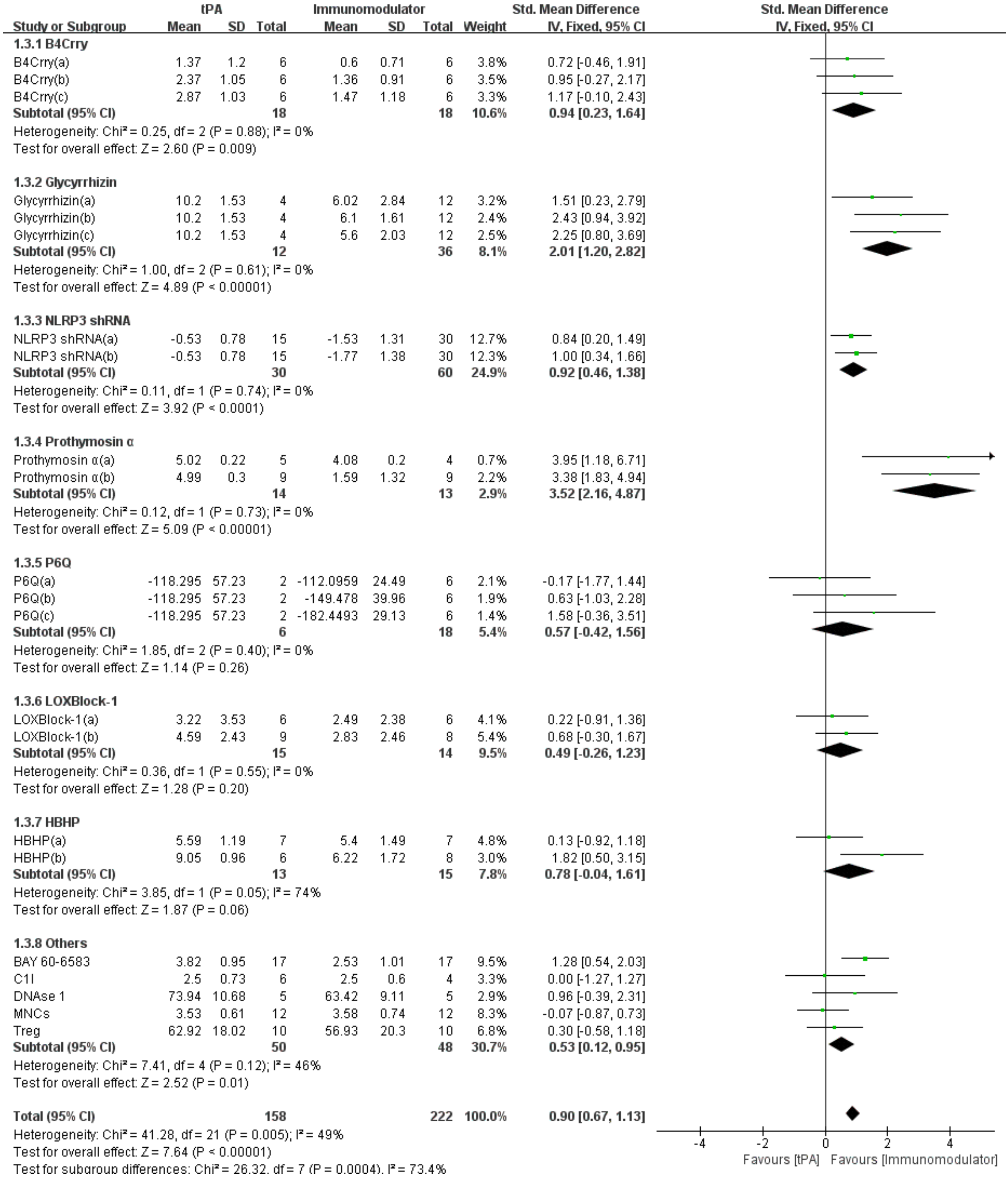

Figure S3. Meta-analysis of immunomodulator on neurobehavioral score. The horizontal lines indicate 95% confidence intervals for individual standardized mean differences (SMD), and the black diamonds indicates global SMD and confidence interval.

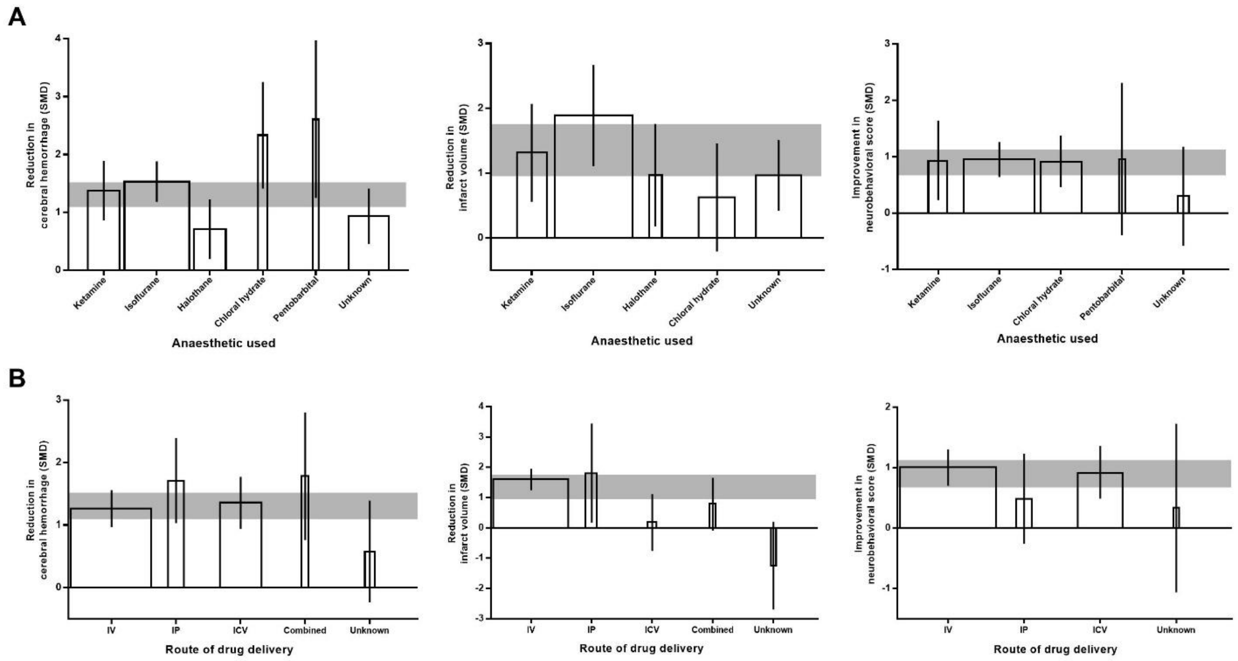

Figure S4. Effect of anaesthetic used (a) and route of drug delivery (b) on the improvement in cerebral hemorrhage, infarct volume, and neurobehavioral score. The width of each bar represent the relative number of animals in that subgroup; vertical error bars represent the 95% confidence interval for the individual estimates; and the horizontal grey bars represent the 95% confidence interval of the pooled estimate of efficacy.

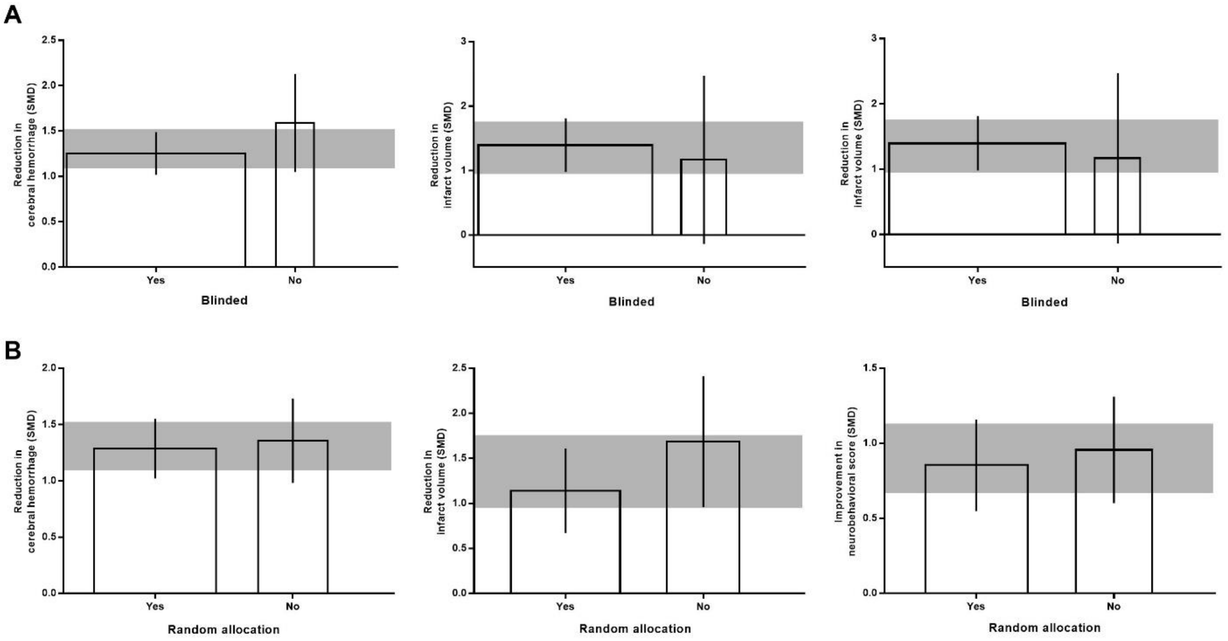

Figure S5. Effect of blinded assessment (a) and random allocation (b) on the improvement in cerebral hemorrhage, infarct volume, and neurobehavioral score. The width of each bar represent the relative number of animals in that subgroup; vertical error bars represent the 95% confidence interval for the individual estimates; and the horizontal grey bars represent the 95% confidence interval of the pooled estimate of efficacy.

Table S1 Study quality score report.

Author	Year	(1)	(2)	(3)	(4)	(5)	(6)	(7)	(8)	(9)	(10)	Aggregate quality score	Journal impact factor (2019)
Alawieh	2020	+	+	+	+	+			+	+	+	8	5.673
Chen	2019	+	+	+	+	+	+		+	+	+	9	5.78
Copin	2008	+	+				+			+		4	4.691
Fan	2013	+		+	+	+	+	+		+	+	8	7.19
Gautier	2009	+	+	+		+	+	+		+	+	8	7.73
Guo	2018	+	+				+			+	+	5	3.37
Halder	2019	+	+		+	+	+		+	+	+	8	4.066
Jin	2018	+	+	+		+	+			+	+	7	7.19
Karatas	2017	+	+		+	+	+			+	+	7	2.733
Lapchak	2007	+				+	+			+		4	2.733
Li	2017	+	+	+	+	+	+		+	+	+	9	5.332
Li	2018	+	+	+		+	+		+	+	+	8	5.793
Liu	2017	+	+	+	+	+	+			+	+	8	7.19
Maeda	2009	+	+				+	+		+		5	2.733

Mao	2017	+		+		+	+		+	+		6	11.337
Murata	2008	+	+				+	+		+	+	6	7.19
Strbian	2007	+	+	+	+	+				+	+	7	23.603
Tan	2019	+	+	+		+	+		+	+	+	8	5.411
Tomasi	2011	+	+	+		+	+			+	+	7	2.74
Yang	2018	+		+		+	+			+	+	6	5.78
Yigitkanli	2013	+	+		+	+	+			+	+	7	9.037
Zhang	2003	+		+		+	+			+		5	7.19

(1) Publication in a peer-reviewed journal
(2) Control of temperature
(3) Random allocation to groups
(4) Allocation concealment (blinded induction of ischemia)
(5) Blinded assessment of outcome
(6) Use of an anesthetic without intrinsic neuroprotective activity (ketamine)
(7) The use of co-morbid animals
(8) Performing a sample size calculation
(9) Compliance with animal welfare regulations
(10) Statement of potential conflicts of interest
